# Supplementary figures and images for: A pan-cancer analysis of prognostic genes
Source: PeerJ. 2016 Feb 16;3:e1499. doi: 10.7717/peerj.1499 (PMC4815555; doi:10.7717/peerj.1499)

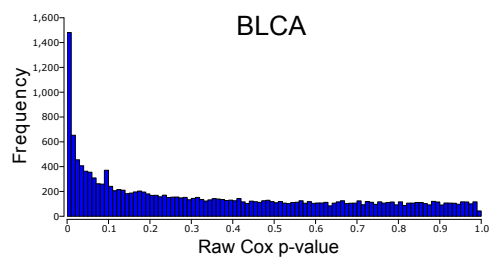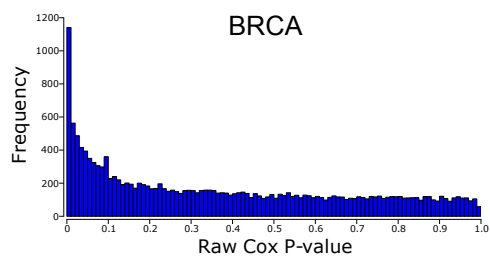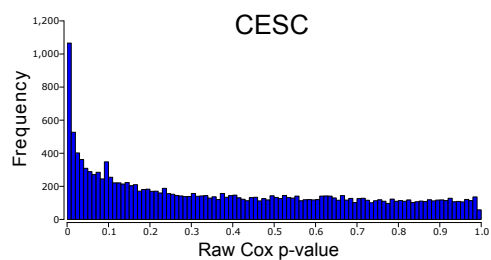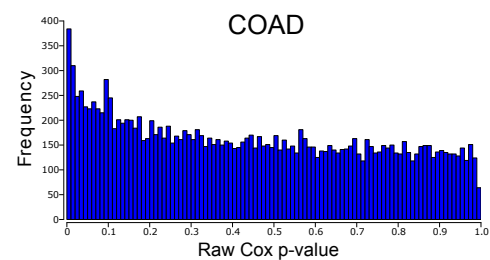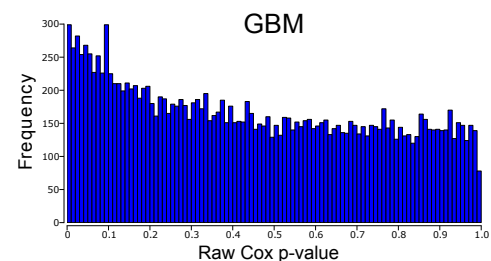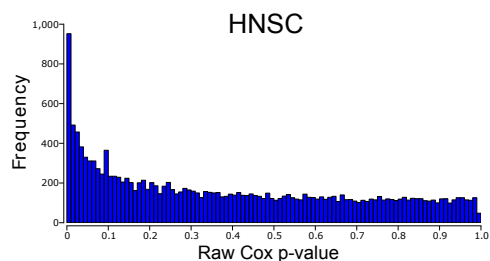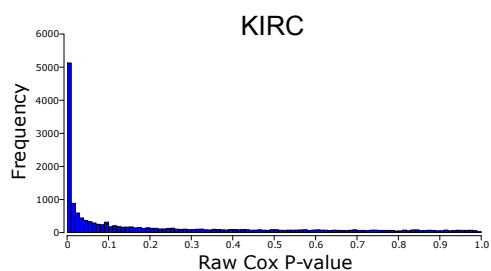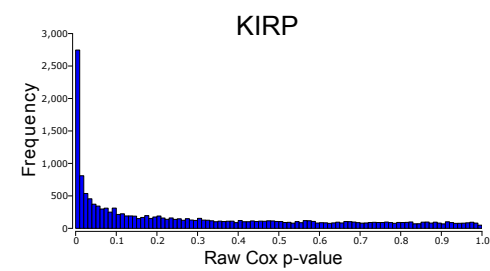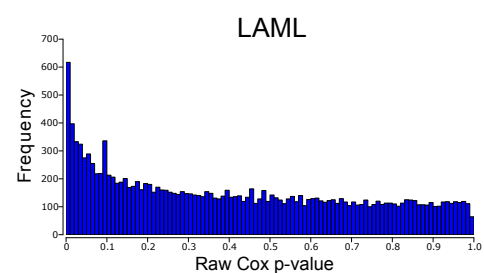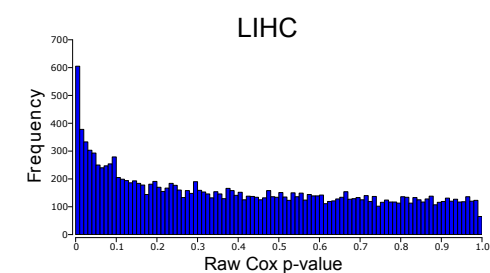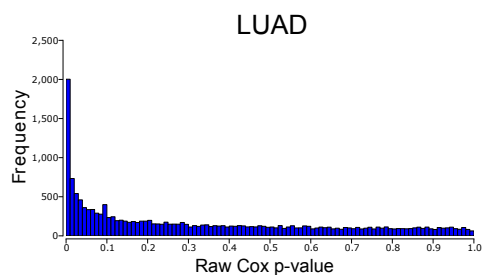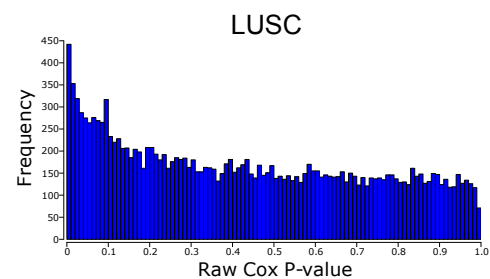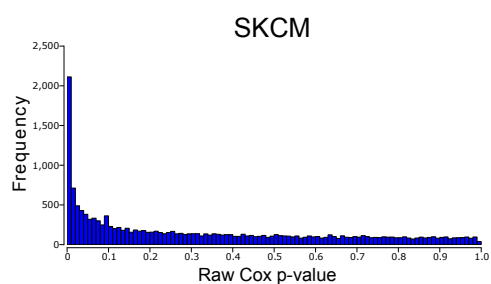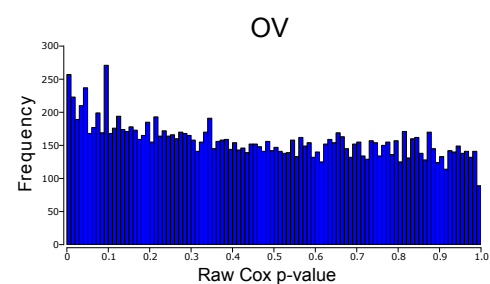

Supplement: Figuge S1 — The distributions of the raw gene p-values from multivariate Cox models are shown for 14 different cancers. [file peerj-03-1499-s004.pdf]
